# The Oral Transfection of *Spodoptera exigua* (Lepidoptera: Noctuidae) Larvae via an Artificial Diet as a Strategy for Recombinant Protein Production

**DOI:** 10.3390/insects16111095

**Published:** 2025-10-25

**Authors:** María Isabel Sáez, Alba Galafat, Pablo Barranco, María Dolores Suárez, Francisco Javier Alarcón, Tomás Francisco Martínez

**Affiliations:** Departamento de Biología y Geología, Universidad de Almería, 04120 Almería, Spain

**Keywords:** artificial diet, chitosan nanoencapsulation, insect larvae, oral transfection, plasmid, recombinant protein

## Abstract

Insects present extraordinary potential for obtaining recombinant proteins, both in terms of the quantity and quality of the synthesized product. This work proposes the use of artificial diets including pDNA as an oral transfection system for the Lepidoptera *Spodoptera exigua*. It is hypothesized that oral transfection can lead to the effective expression of the reporter genes carried in plasmids. Prior to their incorporation into the artificial diet, plasmids (pCMVβ and pEGFP-N2) were protected from inactivation in the digestive tract by chitosan nanoparticulation. The survival of plasmids and their oral uptake by larvae was evaluated, as well as the persistence of pDNA in larvae throughout their ontogeny. The results confirmed that transfection occurred and that pDNA persisted during the ontogeny, even after discontinuing plasmid administration. The transcription of reporter genes was quantified by qRT-PCR, and the results indicate a dose-dependent synthesis of mRNA as the inclusion level of pDNA in diets increased. Moreover, the measurement of the biological activity of the recombinant proteins (β-galactosidase activity and green fluorescence) paralleled the results obtained for gene transcription, also dose-dependently. Therefore, effective oral transfection is feasible in *S. exigua*, provided that pDNA is protected against gut inactivation prior to its incorporation in artificial diets.

## Simple Summary

Recombinant proteins are special proteins made using genetic instructions, and they are widely used in medicines, vaccines, food production, and even agriculture. Among the variety of organisms that can serve to obtain them, insects are valuable natural platforms or “mini-factories” capable of producing recombinant proteins in a safe, massive, and efficient way. In this study, we tested a simple method to deliver plasmid DNA—with instructions for the precise synthesis of proteins—to the larvae of the moth *Spodoptera exigua* by mixing it into their artificial diet. To prevent DNA from being damaged in the insect’s digestive tube, we coated it with chitosan, a natural material, to obtain microscopic particles, and then incorporated it into the diet. The results indicated that larvae successfully absorbed the DNA protected this way and produced the proteins, with higher amounts yielded when more DNA was added. The proteins were easy to detect because they glowed (fluorescence) or showed clear activity. These results show that feeding insects protected DNA could be a practical, economical, and non-invasive way to produce functional proteins useful for a variety of biotechnological applications.

## 1. Introduction

The potential of insects as bio-factories for the synthesis of recombinant proteins, such as hormones, antigens, cytokines, coagulation factors, etc., is virtually unlimited, and therefore, interest in this research field has grown exponentially in recent years [1,2]. Compared to other expression platforms, insects possess remarkable advantages, such as (i) the simplicity of the infrastructure required for rearing and manipulation, which does not involve exigent culture media or fermentation facilities; (ii) the quantitative efficiency of the protein yielded [3]; and (iii) outstanding reproductive potential and short life cycles.

These features and others make insects ideal candidates for mass protein production, that is, for upscaling laboratory processes to the industrial level [1,4,5]. But beyond any economic or productive considerations, it is likely that the most crucial concern for any expression system is related to the quality of the recombinant protein synthesized. In this regard, it has been described that entomo-expressed proteins keep their structure and biological activity, such as antigenicity, in several species of domestic animals [4,6,7,8], owing to the fact that insect cells possess efficient post-translational pathways [9], including glycosylation, phosphorylation, disulfide bond formation and protein processing, required for the biological activity of many complex proteins [10].

Besides the abovementioned advantages, however, a common limitation of any expression system is related to the need to achieve an efficient, controllable and quantifiable transfection of the construct encoding for the specific protein required, which determines the choice of the most adequate expression vector for a given platform. When it comes to considering insects, the most potent and therefore widely used vector is the baculovirus expression vector system (BEVS), based on transformed Baculovirus as a carrier of foreign genes of interest and insect cells as the host for gene amplification and recombinant protein expression. Baculoviruses are insect pathogens that can cause death in lepidopteran, dipteran and hymenopteran larvae, but their interest as an expression system in cell culture is related to the fact that the baculovirus double-stranded DNA genome can be easily modified to incorporate genes of interest via homologous recombination [11]. Therefore, the BEVS is the most well-known and widespread used system for the large-scale production of complex proteins in insect cells, based on its ability to transfect insect cells quite specifically and produce a high yield of protein [12] with post-translational modifications and based on its high safety and regulatory compliance [13]. In addition, the use of multiple promoters makes possible the expression of several proteins simultaneously in a single infection. The most used lepidopteran insect cell lines are Sf21 and Sf9 derived from *Spodoptera frugiperda* and the BTI-TN-5B1-4 cell line (High Five™) derived from *Trichoplusia ni* [14].

As a result of the mentioned advantages, commercially available vaccines produced in insect cells exist for several different indications in both human (e.g., human papillomavirus and influenzavirus) and veterinary medicine (e.g., classical swine fever, porcine circovirus, porcine parvovirus) [10,15].

However, even if insect cell platforms enable a feasible scale up of protein production, it is also safe to say that the demanding cell culture conditions, including expensive culture media and the use of bioreactors, which increases the production costs, are disadvantages to producing recombinant protein at the industrial level. In addition, although the growth rate of insect cells is higher than mammalian cells, it is still lower when compared to yeast or bacteria, and production requires more expensive culture media and longer times than those required for microbial systems.

In this context, an additional and complementary strategy for the mass production of recombinant proteins could involve whole insect larvae as an expression platform, rather than cultured insect cells, since larvae are easier to mass rear and do not require complex cell culture facilities. The advantages related to the high protein expression levels and the post-translational modifications of complex proteins mentioned for insect cells are extensive for insect larvae, although further processing to purify recombinant products from insect larvae is likely more complex than that established for cultured cells. This platform might allow for heterologous protein expression with no need to genetically modify the host genome, taking into account that BEVS transfection is not only limited to insect cell cultures but also to insect larvae or pupae, which are susceptible to Baculovirus infection (in fact, they have been used in pest control owing to their ability to cause death in lepidopteran, dipteran and hymenopteran larvae). *Autographa californica* multiple nucleopolyhedrovirus (AcMNPV) and *Bombyx mori* nucleopolyhedrovirus (BmNPV) are the most extensively used vectors in the BEVS to produce heterologous proteins in insect larvae.

However, the viral transfection of whole larvae using Baculovirus involves a range of limitations, the most obvious being the inevitable death of the larvae, with no control over the timing. In this context, transfection by means of inert vectors might offer a viable alternative, as it could overcome the limitations associated with the BEVS in whole larvae.

The most widely used insect species in basic research as bioreactors for recombinant protein production belong to the order Lepidoptera. For instance, human alpha-interferon (IFN-alpha) was produced four decades ago in *Bombix mori* larvae [16], but a range of other recombinant proteins have been expressed in this species since then, such as carp reovirus capsid proteins [17] or bovine papillomavirus antigens [18]. Research has also been conducted on other lepidopteran species with successful results, such as *Spodoptera frugiperda* (rotavirus antigens [19]), *Heliothis virescens* (influenza virus hemagglutinins [20]), *Rachiplusia nu* (bovine viral diarrhea virus glycoprotein [21]), and *Trichoplusia ni* (West Nile virus envelope protein [4]; influenza virus hemagglutinins [5]; rotavirus antibodies [7]; and human growth factors [1]).

Unlike the silkworm, some lepidopteran species can be reared on artificial diets, a fact that might enable the oral administration of substances of interest. Additionally, polyvoltine species, capable of multiple generations per year, offer a significant advantage for large-scale protein production compared to *B. mori* due to their potential for continuous rearing.

The expression of recombinant proteins in insects via oral transfection with pDNA was proposed several decades ago [20,21,22]. However, to our knowledge, this approach has not yet proven viable, at least on an industrial or semi-industrial scale. Among the various prerequisites for successful oral delivery, two of them are worth mentioning as the main limitations: (i) the selected insect species must be able to accept an artificial diet and (ii) the recombinant plasmid must remain stable during its passage through the digestive tract of larvae.

Insects are endowed with potent intestinal nuclease activity [23,24], making it unlikely that significant amounts of orally administered naked pDNA can survive passage through the digestive tract. Without this prerequisite, the plasmid cannot be internalized by the larvae, preventing effective transfection and subsequent gene expression. One potential strategy to overcome this limitation involves the use of encapsulation techniques. Although this approach remains virtually unexplored in insect larvae, except for the delivery of nanoparticulated dsRNA primarily for pest control [25], it has proven successful in other animals to protect biomolecules during the gastrointestinal transit, such as fish [26,27,28,29,30,31] and murine models [32,33]. This strategy may also be of interest for achieving the efficient oral delivery of pDNA in insect larvae. To our knowledge, no studies have been reported that specifically investigate the successful oral delivery of plasmids in Lepidopteran larvae or in other insect models.

Considering the abovementioned antecedents, it is hypothesized that oral transfection of *Spodoptera exigua* larvae with plasmid DNA incorporated into an artificial diet can result in the effective expression of reporter genes, provided that plasmids have previously been protected by nanoencapsulation.

## 2. Materials and Methods

### 2.1. Animals

*Spodoptera exigua* specimens used in the assays were obtained from a generation grown in a laboratory with an artificial diet since its initiation. The parentals were obtained from wild female individuals captured using light traps, confined in paper oviposition chambers for egg laying and fed with honey water solution on cotton wool. Egg masses on paper were cut daily and transferred to hatching chambers for three days. Most eggs hatched in two days. One-day larvae were moved to 25 mL vials containing a small cube of freshly prepared artificial diet.

This study does not involve any animal species or research activity that falls under the scope of European Union (Directive 2010/63/EC) legislation on the protection of laboratory animals.

### 2.2. Artificial Diet for Spodoptera exigua Larvae

An artificial diet specially developed for this species in the Applied Entomology laboratory (Universidad de Almería, Spain), adapted from Poitout and Bues [34] and described by Cabello et al. [35], was used. The diet was made up of corn flour, wheat germ, and brewery yeast, embedded in microbiological agar, in a 2:2:2:1 ratio. Once dissolved in boiling distilled water, agar was warmed up to 50 °C, and then the rest of the ingredients were added and mixed thoroughly. Prior to solidification, the mixture was aliquoted in bacteriological plates, in an approximately 5 mm thick layer, and then stored at 4 °C until further use. Each batch of unused diet was discarded after two weeks.

### 2.3. Plasmid Vectors and Reporter Genes

Two pDNA-based eukaryotic expression vectors were used in this study (both from Clontech Laboratories, Inc., Palo Alto, CA, USA), the plasmid pCMVβ (GenBank #U02451), integrating the *lacZ* reporter gene encoding for the *E. coli* β-galactosidase enzyme (EC 3.2.1.23, β-gal), and plasmid pEGFP-N2 (GenBank #U57608), encoding for an enhanced jellyfish green fluorescent protein gene, *EGFP*. A remarkable advantage of these constructions is represented by the possibility of measuring the biological activity of the expression products by means of an enzymatic assay (β-galactosidase enzyme activity for pCMVβ) or a fluorometric quantification (green fluorescence for pEGFP-N2).

**Figure 1:**
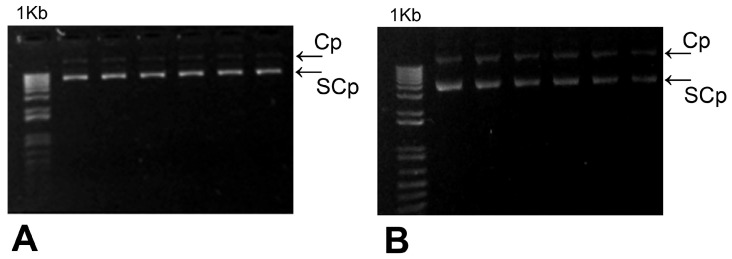
Electrophoretic separation in 1% agarose gels of pCMVβ (**A**) and pEGFP-N2 (**B**) plasmids purified from transformed *E. coli* DH5α. Cp and SCp stand for circular and supercoiled plasmid isoforms, respectively. 1 Kb: molecular mass marker.

Host *E. coli* (DH5α) cells were transformed independently with pCMVβ and pEGFP-N2 and then grown in LB medium containing 100 μg mL^−1^ ampicillin or kanamycin, respectively, as selection markers for transformed colonies. Bacterial biomass was collected, and plasmid DNA was purified with a commercial kit (Gigaprep Gen Elute HP, Sigma-Aldrich, St. Louis, MI, USA), according to the manufacturer’s instructions, and then quantified (Nanodrop 2000, Thermo Fisher, Waltham, MA, USA). The quality and integrity of the extraction were assessed by electrophoretic separation in 0.8% (*w*/*v*) agarose gels and stained with SYBR Green^©^ (Biomol, Madrid, Spain), and specifically, the presence of supercoiled pDNA, characterized by higher electromobility in gels compared to circular forms, was verified as described in Tian et al. [27], as shown in Figure 1.

### 2.4. Chitosan–pDNA Nanoparticles

The preparation of chitosan nanoparticles was carried out according to the protocol described in Sáez et al. [30] with some modifications. Briefly, a 0.5% chitosan solution in 25 mM sodium acetate, pH 5.5, was prepared. Separately, plasmid solutions containing 100 μg mL^−1^ DNA in 0.5% (*w*/*v*) sodium sulfate buffer was made. Equal volumes of both solutions were immediately mixed and vortexed intensely for 1 min and then allowed to stand for 30 min at room temperature. Finally, nanoparticles were recovered by centrifugation (12,000× *g*, 10 min). With the purpose of determining the proportion of DNA loaded in chitosan–pDNA nanoparticles, the encapsulation efficiency (EE) of the complex was calculated from the total amount of DNA added to the formulation mixture and the DNA that was not entrapped in nanoparticles, according to the equation EE(%) = [(C_1_ − C_2_)/C_1_] × 100, where “C_1_” is the concentration of DNA in the initial solution, and “C_2_” is DNA concentration in the supernatants of the encapsulation medium after removing chitosan–pDNA nanoparticles by centrifugation. DNA concentration was determined in all cases by measuring UV absorbance at 260/280 nm (Nanodrop 2000, Thermo Fisher). EE was higher than 95% in all the assays.

### 2.5. Assessment of Larvae Regarding the Integrity of pDNA Orally Delivered

Prior to the elaboration of the experimental diets containing different concentrations of nanoencapsulated plasmids, a preliminary experiment was carried out, in which the integrity of naked and nanoparticulated pDNA included in diets after oral ingestion by larvae was assessed. With this aim, 30 larvae (L3 instar) were distributed in three experimental batches (10 larvae each), and the animals were fed with the following diets: CT (without pDNA), naked-pDNA (containing 50 μg of the naked plasmid pCMVβ g^−1^ diet) and nano-pDNA (containing 50 μg of the nanoparticulated pCMVβ g^−1^ diet). In order to ensure that each larva ingested the same amount of plasmid during the feeding period, they were arranged in individual containers (25 mL vials in which a square of 1 × 1 cm was cut out of the lids and then covered with fine mesh). Each larva was housed with 0.2 g of the respective diets until the portion was completely ingested. Individual confinement was intended to ensure the complete ingestion of the set amount of pDNA per larva. Then, the larvae were stored at −20 °C until their use for the extraction of DNA and detection by a conventional PCR of specific sequences of plasmid pCMVβ in larval extracts, as will be explained below.

### 2.6. Persistence of pDNA in Larvae Throughout Their Ontogenetic Development

A second in vivo assay was performed with *Spodoptera exigua* larvae in order to assess whether the discontinued administration of the pDNA via diet would enable the persistent presence of the plasmid in larval tissues throughout the rest of the larval life cycle (including the adult stage) or, on the contrary, whether the presence of pDNA would fade, and therefore, it would be necessary to continuously re-feed the insects to maintain certain expression levels. Based on the results of the previous trial, it was confirmed that pDNA intended for oral administration must be protected by nanoencapsulation, and hence, plasmids were prepared in this manner prior to their inclusion in all the diets.

For this purpose, 2 batches of recently hatched larvae (80 individuals per batch) (L1 instar) were prepared as follows: (a) a control group (CT, without pDNA in the diet) and (b) a nano-pDNA batch, in which a nanoparticulated pCMVβ plasmid was added to the diet (50 μg pDNA g^−1^ diet). Larvae were mass reared, and the respective diets were offered to L1-stage larvae until reaching the L3 stage. Then, all larvae received, during the rest of their ontogenetic development, the control diet (without plasmids). After each new molting process (L4, L5, pupa, and imago), 10 individuals were removed from each batch and stored at −20 °C for subsequent PCR determinations.

### 2.7. Oral Transfection with Different Concentrations of pDNA

A third in vivo experiment was carried out, in which three different concentrations of plasmids in the diets were assessed, with the aim of determining the influence of the plasmid inclusion level in diets on the subsequent quantification of the expression of the reporter genes after oral transfection.

Four experimental batches of L3 larvae (20 specimens per batch, 80 larvae in total) were laid out. The first lot (control, CT) was fed on the standard experimental diet prepared as described, which did not include pDNA. The other three batches (D25, D50, and D100) were fed with diets including the two nanoencapsulated plasmids (pCMVβ and pEGFP-N2) simultaneously in the experimental diet (25, 50, and 100 μg total pDNA g^−1^ diet, respectively). Therefore, diets D25, D50, D100 contained, respectively, a final concentration of 12.5, 25 and 50 μg pDNA g^−1^ of each individual plasmid. Larvae were arranged in individual 25 mL vials as described previously, and once the 0.2 g portion of the diet was ingested, all larvae were re-fed for three additional days with the CT diet (without pDNA). Therefore, individual larvae ingested 2.5, 5 and 10 μg of each individual plasmid from D25, D50 and D100 diets, respectively (double the amount of total pDNA since the diets included both plasmids simultaneously). At the end of this period, 6 larvae per treatment were immersed in RNAlater for further RNA extraction, whereas the remaining were stored at −20 °C until their use for the rest of the determinations.

### 2.8. Plasmid Detection in Larvae by Conventional PCR

After oral transfection, five larvae were taken from each experimental batch in order to identify specific sequences of both plasmids (pCMVβ and pEGFP-N2) by conventional PCR after DNA extraction from larvae with a purification kit (DNeasy Tissue Kit, Qiagen, Hilden, Germany), according to the manufacturer’s instructions.

The extracted DNA was used as a template in conventional PCR reactions with specific primers (Table 1) for the amplification of different sequences of both plasmids. The constitutive gene of β-actin was used as the control in all amplification reactions (primer pair forward: 5′-ACATGGAAATCTGTGCACCACA-3′; reverse: 5′-ACAGCTTTTCTTTGATGTCGCGAA).

In the case of pCMVβ, PCR cycling conditions included a 5 min activation step, followed by 36 cycles of 1 min at 95 °C, 2 min at 50 °C, and 1 min at 72 °C, with a final extension step at 72 °C for 10 min; and the amplification process was finalized at 4 °C. For pEGFP-N2, slightly different conditions were carried out; namely, the second step of each of the 36 cycles consisted of 1 min at 58 °C, with all other conditions of the PCR reactions being identical.

The final concentrations for all PCR components in a 25 μL volume were 10 nM of each dNTP, 4 pM of each pCMVβ or pEGFP-N2 specific primer, 20 μg µL^−1^ of extracted genomic DNA, 0.5 units GoTaq-Flexi-DNA polymerase (Promega Biotech Ibérica, SL, Madrid, Spain) and 5 µL Taq buffer pH 8.5 (5X Green GoTaq-Flexi-Buffer; Biomol, Madrid, Spain) supplemented with 2 mM MgCl2. PCR products were separated in 0.8% (*w*/*v*) agarose gels containing SYBR Green^©^ (Biomol, Madrid, Spain) and exposed to UV light (λ 312 nm) using an ETXF transilluminator (Vilber-Lourmat, Madrid, Spain). One Kb Plus DNA ladder (Invitrogen, Waltham, MA, USA) was used as the molecular mass marker.

### 2.9. Quantification of Gene Expression in Larvae

#### 2.9.1. RNA Extraction and cDNA Synthesis

RNA was isolated from 5 larvae per dietary treatment, using a commercial kit (Genejet RNA Purification kit, Thermo Scientific, Waltham, MA, USA), according to the manufacturer’s instructions. The amount of RNA extracted was determined (Qubit^©^ 4 fluorometer, Thermo Scientific, Waltham, MA, USA), and reverse transcription was performed using a commercial kit for this purpose (First Strand cDNA Synthesis Kit, Thermo Scientific, Waltham, MA, USA), starting from an amount of 500 ng total RNA as the template. Once the corresponding cDNA was obtained, a microliter of the solution was used as a template in the qPCR assay to quantify the degree of transcription of each gene.

#### 2.9.2. Quantification of Reporter Gene Expression

Specific pairs of primers for the quantification of the relative gene expression of *lacZ* and *EGFP* genes, encoding for bacterial β-galactosidase activity, and the green fluorescent protein, respectively, were designed in our laboratory (Table 2). For standardization, larval samples were analyzed in parallel with three constitutive reference genes: ribosomal protein L10 (*rsp10*, GenBank #ABX54738.1), glyceraldehyde 3-phosphate dehydrogenase (*gadph*, GenBank #AEJ38217.1) and *S. exigua* actin (*act*, GenBank # AEJ38214.1).

The qPCR reactions were performed on a 1000 Touch™ thermal cycler (BioRad, Madrid, Spain) with the CFX96™ optical module (BioRad, Madrid, Spain) for fluorescence measurements. Amplification reactions were performed in triplicate in 96-well plates with a final volume of 10 μL per well. The mixture contained 5 μL of GoTaq© qPCR Master Mix (Promega, Madison, WI, USA), 0.5 μL of direct and reverse primers (10 μM), 1 μL of cDNA and 3 μL of nuclease-free water. For the *lacZ* gene, an initial activation of Taq polymerase at 95 °C for 3 min was set, followed by 40 cycles of 95 °C for 15 s and 60 °C for 60 s. For the *EGFP* gene, PCR was performed at 95 °C for 10 min, then 40 cycles at 95 °C for 10 s, and 60 °C for 20 s. Finally, for the reference gene (*rsp10*, *gadph* and *act*) reactions were incubated at 90 °C for 5 min followed by 40 cycles of 95 °C for 10 s, 60 °C for 10 s and 72 °C for 15 s. Amplification threshold (Cq) values greater than 40 were considered negative. The efficiency of each primer was assessed using a series of two-fold serial dilutions of cDNA. Primer efficiencies ranged from 99% to 102%, so the variations were not considered to significantly influence gene expression quantification. Therefore, the relative mRNA expression levels were calculated by the procedure 2^(−ΔΔΔCq)^ according to Livak and Schmittgen [36], normalized with a geometric average of the three reference genes and related to larvae in each control group (without pDNA ingestion).

### 2.10. Quantification of the Biological Activity of Entomo-Expressed Recombinant Proteins

#### 2.10.1. Quantification of β-Galactosidase Activity in Larval Extracts

The possible expression in larval tissues of the bacterial lacZ gene (encoding for the bacterial β-galactosidase enzyme) integrated in expression vector pCMVβ was measured by an enzymatic assay based on [37], using *o*-nitrophenyl-β-galactopyranoside (ONPG) as the substrate for the β-galactosidase enzyme (β-gal). Briefly, five larvae of each dietary treatment were homogenized (200 mg mL^−1^) in 0.1 M Tris-HCl, pH 7.0, and 100 μL of the extract was mixed with 100 μL of 4 mg mL^−1^ ONPG in 0.1 M Tris-HCl, pH 7.0, 50 μL 1 M β-mercaptoethanol, and 450 μL of a solution containing 0.1 M sodium phosphate, 10 mM KCl, 1 mM MgSO4, pH 8.0. The mixture was incubated for 24 h at 37 °C and then absorbance (λ 420 nm) was measured spectrophotometrically (Multiskan EX, Thermo Fisher, Waltham, MA, USA) with the aim of quantifying the amount of *o*-nitrophenol released due to the hydrolysis of ONPG by the heterologous β-gal enzyme. A β-galactosidase activity unit (U) was defined as the amount of enzyme that hydrolyzed 1.0 μmole of ONPG to *o*-nitrophenol and D-galactose per min under the specified assay conditions. A commercial β-galactosidase enzyme (from *E. coli*, grade VIII, Sigma Aldrich, Madrid, Spain) was used as the positive control in the assays.

#### 2.10.2. Quantification of Green Fluorescence in Larval Extracts

Similarly to what was described for β-gal enzyme activity, larval extracts (5 larvae per dietary treatment) were prepared for each treatment (200 mg mL^−1^), and possible fluorescence in tissues owing to *EGFP* expression was measured fluorometrically (Fluoroskan Ascent, Thermo Fisher, Waltham, MA, USA), using λ 488 nm excitation and λ 532 nm emission wavelengths, respectively.

### 2.11. Statistical Analysis

Statistical analysis was carried out to determine if there were significant differences in the quantification of the copy number of the specific sequences of both genes and the quantification of β-galactosidase and EGFP activities measured by spectrophotometry and fluorimetry, respectively, in the extracts of the larval tissues. For this, the one-factor analysis of variance method was used, and a comparison of means was performed using the LSD (Fisher’s Least Significant Difference) test with SPSS 25 software of IBM Corporation Inc. (Armonk, NY, USA). A significance level of 95% (*p* < 0.05) was established for the determination of statistical differences.

## 3. Results

### 3.1. Detection of Specific Sequences of pDNA in Orally Transfected Larvae

**Figure 2:**
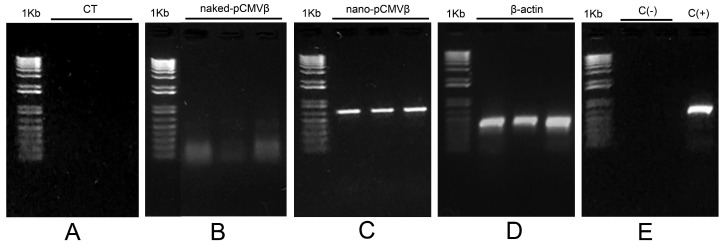
Electrophoretic separation of PCR products after the amplification of specific sequences of plasmid pCMVβ from *Spodoptera exigua* larval extracts (L3 instar). Larvae were fed with the following experimental diets: CT ((**A**) without pDNA), naked pDNA ((**B**) containing 50 μg g^−1^ naked pCMVβ) and nano-pDNA ((**C**) containing 50 μg g^−1^ chitosan-nanoparticulated pCMVβ). β-actin was used as the reference gene (**D**). Positive (the pCMVβ plasmid, C+) and negative (distilled water, C−) controls are shown in (**E**). 1 Kb: molecular mass marker.

The results of the preliminary experiment aimed at assessing the possible presence of pDNA in larval extracts after its inclusion in the experimental diets are shown in Figure 2.

The electrophoretic separation of the PCR products resulting from amplifying specific sequences of the pCMVβ plasmid in larval extracts confirmed the presence of the recombinant plasmids only in larval tissues of *Spodoptera exigua* fed with diets including chitosan-nanoparticulated pDNA (Figure 2C). The presence of the specific amplicons was not detected in the control group, in those fed with the diet without pDNA (CT-no-pDNA lanes, Figure 2A) or in larvae fed with naked pDNA (Figure 2B). Negative (distilled water, C−) and positive (pCMVβ plasmid, C+) controls were also considered in the experiment (Figure 2E). Constitutive β-actin, used as a reference gene, was amplified in all samples (Figure 2D).

**Figure 3:**
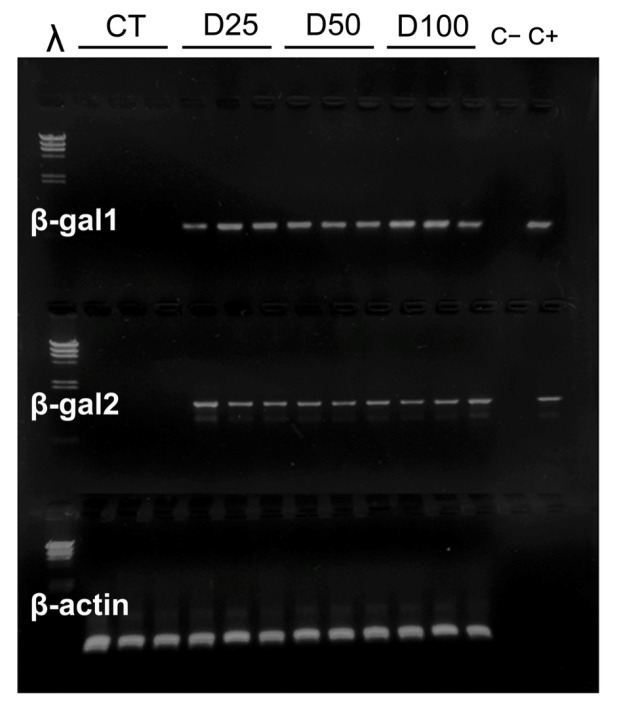
Electrophoretic separation in 1% agarose gels of specific PCR products of pCMVβ amplified from *S. exigua* larval extracts. CT: control batch without pDNA in diet. D25; D50; D100: experimental diets including increasing amounts of chitosan-nanoparticulated pCMVβ. λ: molecular mass marker. β-gal1, β-gal2, and β-actin are amplicons from primer pairs according to Table 1.

**Figure 4:**
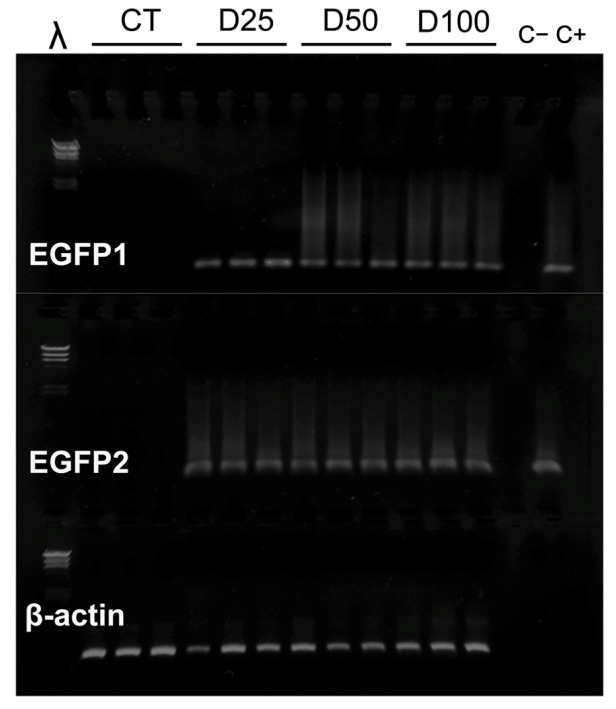
Electrophoretic separation in 1% agarose gels of specific PCR products of pEGFP-N2 amplified from *S. exigua* larval extracts. CT: control batch without pDNA in diet. D25; D50; D100: experimental diets including increasing amounts of chitosan-nanoparticulated pEGFP-N2. λ: molecular mass marker. EGFP1, EGFP2 and β-actin are amplicons from primer pairs according to Table 1.

Consequently, only protected pDNA was contemplated for the rest of the experiments, and thus, plasmids were nanoparticulated prior to their incorporation into the experimental diets. The results of the detection of specific sequences of pCMVβ and pEGFP-N2 plasmids in larval extracts (L3 instar) after their inclusion in the experimental diets at different concentrations (CT, D25, D50, and D100, as explained in M&M) are shown in Figure 3 (pCMVβ) and Figure 4 (pEGFP-N2). Amplification was observed in all samples, irrespectively of the pDNA concentration, of *S. exigua* extracts and in the positive control with pDNA (C+), but not in distilled water (lane C−).

### 3.2. Detection of Specific Sequences of pDNA During the Ontogenetic Development

**Figure 5:**
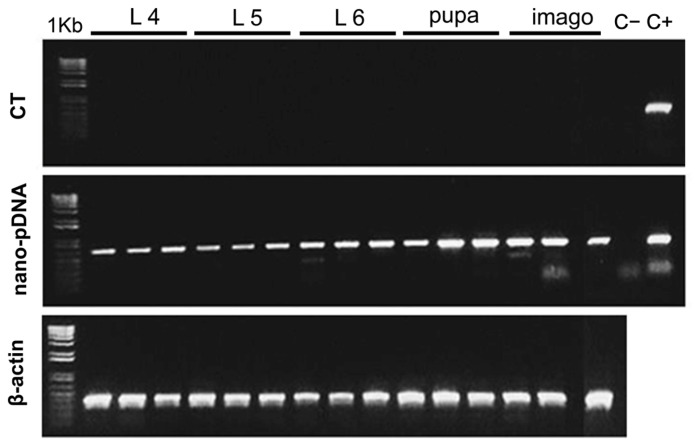
Electrophoretic separation of PCR products after the amplification of specific sequences of the pCMVβ plasmid from *S. exigua* extracts of the control (CT) and orally transfected larvae with 50 µg of the chitosan-nanoparticulated pDNA g^−1^ diet (nano-pDNA) throughout different molting stages. Larvae were fed with the pDNA-enriched diet for a limited period of time (prior to molting to L3) and then the control diet (without pDNA) during the rest of the ontogenic stages up to the adult phase. 1 Kb: molecular size marker.

The results of the detection of specific fragments of the pCMVβ plasmid during the ontogeny of *S. exigua* larvae are shown in Figure 5. These larvae ingested a diet enriched with 50 μg pDNA g^−1^ from hatching to L3, and afterwards, they were fed on the plasmid-free (control, CT) diet until reaching the adult stage. As observed, pDNA persisted and could be amplified in larval tissues throughout the following instars, in pupae, and even in the stage of imago since specific sequences of pCMVβ were detected.

### 3.3. Relationship Between pDNA Inclusion Level in Diets and Reporter Gene Expression in Larvae

**Figure 6:**
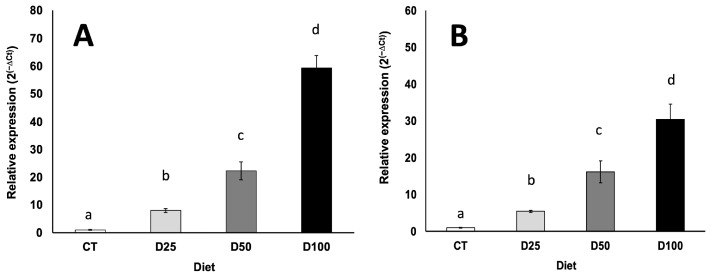
Influence of the concentration of chitosan-nanoparticulated pCMVβ (**A**) and pEGFP-N2 (**B**) plasmids included simultaneously in the experimental diets on the expression level of *lacZ* (**A**) and *EGFP* (**B**) reporter genes in *S. exigua* larvae transfected orally. Number of copies (2^(−ΔCt)^). CT, D25, D50 and D100 stand for 0 (control), 12.5, 25 and 50 micrograms of each plasmid g^−1^ diet (double these quantities for total pDNA). Different lower-case letters in columns indicate significant differences attributable to pDNA concentration in the experimental diets (*p* < 0.05).

Once the specific sequences of both plasmids were detected in larval extracts, the next step consisted of the quantification of the expression of the reporter genes of both plasmids (by qRT-PCR) in such extracts. The number of copies quantified *S. exigua* larvae fed with the experimental diets increased in parallel with the increasing inclusion level of chitosan-nanoparticulated pDNA in the experimental diets (Figure 6A for pCMVβ and Figure 6B for pEGFP-N2). This finding confirmed a clear dose dependence and proportionality between the concentration of pDNA nanoparticles in the diets and the number of copies of both reporter genes (*lacZ* and *EGFP*) quantified in larval tissues (R^2^ values of 0.882 and 0.953 for pCMVβ and pEGFP-N2, respectively)

### 3.4. Quantification of the Biological Activity of the Entomo-Expressed Protein in Larvae

**Figure 7:**
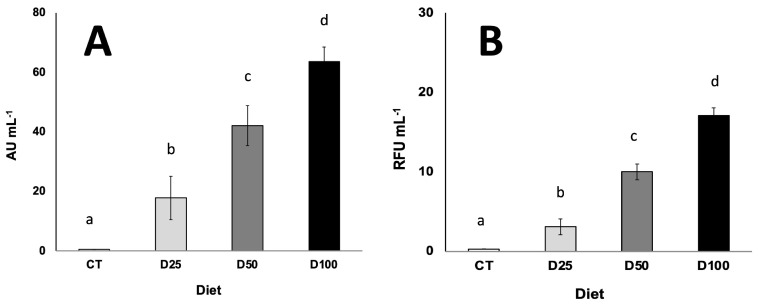
Quantification of β-galactosidase enzyme activity (**A**) and green fluorescence (**B**) measured in extracts of *S. exigua* larvae transfected orally with diets containing increasing amounts of chitosan-nanoparticulated pCMVβ (**A**) and pEGFP-N2 (**B**) plasmids. CT, D25, D50 and D100 stand for 0 (control), 12.5, 25 and 50 micrograms of each plasmid g^−1^ diet (double these quantities for total pDNA). AU**s**: activity units. RFU**s**: relative fluorescence units. Different lower-case letters in columns indicate significant differences attributable to pDNA concentration in the experimental diets (*p* < 0.05).

In order to assess whether the presence of the specific sequences of plasmids pCMVβ and pEFGP-N2 in larvae was also associated with the expression of both reporter genes in *S. exigua* larvae, the biological activity of de novo recombinant proteins was measured. As shown in Figure 7A, it was possible to detect and quantify β-galactosidase activity in extracts prepared from orally transfected larvae, whereas in the control individuals, β-galactosidase activity was not found. Similarly, Figure 7B shows the fluorescence measured because of the newly synthesized EGFP protein after the oral transfection of larvae with nanoparticulated–pEGFP-N2 in the diets. As observed in Figure 7, the higher the inclusion level of both plasmids in the experimental diets, the higher the biological activity measured in larvae in a clearly proportional manner (R^2^ values of 0.996 and 0.967 for β-gal activity and green fluorescence, respectively).

## 4. Discussion

In parallel with the expansion of the industrial rearing of insects, primarily for biomass production for food and feed, there is growing interest in their use as expression platforms for the mass production of recombinant proteins. Insects offer several advantages over other expression systems, many of which have been previously mentioned and will not be further discussed. The abundant literature about insect cell transfection and recombinant protein synthesis in bioreactors lies in the fact that Baculovirus viral vector is a well-established and efficient transfection method aimed at introducing genes encoding for heterologous protein, particularly antigens and therapeutic molecules [1,2,38].

To be viable for mass production, insect species must be able to be reared on artificial diets, a fact that opens new opportunities for the oral delivery of nucleic acids, a promising route of transfection for producing entomo-expressed recombinant proteins. Oral delivery offers several practical advantages, such as a non-invasive and convenient application, the lack of special biosafety limitations, and certain control of the dosage [39].

The concept of oral delivery of nucleic acids in insects is not new since it has been explored for agronomic purposes, primarily for pest control via the silencing of genes by means of dsRNA. Early studies relied on injection or soaking to deliver dsRNA, but subsequent research has demonstrated the feasibility of RNAi oral delivery. Tian et al. [40] fed *Spodoptera exigua* larvae with a diet enriched with E. coli expressing dsRNA targeting the chitin synthase A gene (SeCHSA). The results indicated that effective transfection occurred, which resulted in reduced SeCHSA expression, altered development, and increased mortality. The feasibility of the direct inclusion of dsRNA in the artificial diet has also been investigated, and thus, Turner et al. [41] fed larvae of the lepidopteran *Epiphyas postvittana* with dsRNA against two genes coding for an intestinal esterase and a pheromone receptor, and the results confirmed the presence of specific RNAi in larvae.

Whyard et al. [42] used artificial diets containing dsRNA against ATPase-encoding genes in three species of insects, *Tribolium castaneum*, *Manduca sexta* (both solid diets) and *Acyrthosiphon pisum* (a liquid diet) and reported 50 to 75% mortality. Surakasi et al. [43] reported that *S. exigua* larvae fed with cabbage leaves previously treated with a solution of dsRNA against an integrin of the intestinal epithelium led to increased mortality. These results demonstrate the potential of oral RNAi strategies in insect pests and align with previous findings indicating the resistance of dsRNA to ribonuclease degradation [44].

However, not all attempts in this regard have been successful, and thus, Whyard et al. [42] were unable to achieve RNAi in larvae of four species of *Drosophila* fed with dsRNA. This discrepancy may well be attributed to interspecific differences in the activity of dsRNA-degrading enzymes, as proposed by Peng et al. [45], which could explain why, in general, injected dsRNA results in stronger RNAi effects than oral delivery.

In contrast to the studies focusing on gene silencing via the oral delivery of nucleic acids, the aim of the present work was the oral administration of pDNA encoding genes of interest for recombinant protein expression. Unlike dsRNA, pDNA is highly susceptible to degradation in the insect digestive tract, which is rich in nucleases that compromise the stability and uptake of exogenous DNA. Moreover, for pDNA to keep its functionality, it must retain not only its primary (nucleotide sequence) and secondary (double-strand) structures but also its tertiary configuration (specifically the “supercoiled” isoform). In other words, the lineal or relaxed circular isoforms of pDNA are ineffective for gene expression. Consistent with these limitations, when naked pDNA was included in artificial diets in our study, neither plasmid-specific sequences could be recovered from larvae (Figure 2B), nor was the in vivo expression of the reporter genes observed in the insects.

In contrast, when pDNA was complexed into nanoplexes and delivered orally via the artificial diet, exogenous pDNA was detected in larval tissues. The PCR amplification of sequences specific to both pCMVβ and pEGFP-N2 constructs confirmed the presence of plasmid DNA in *S. exigua* extracts (Figure 2C, Figure 3 and Figure 4). These findings indicate that a significant amount of pDNA successfully overcame enzymatic degradation by intestinal nucleases existing in the larval digestive tract [23,24,46]. This supports the need to protect the plasmid constructs by means of encapsulation techniques prior to dietary delivery in order to preserve their structural integrity and biological functionality. Although still scarcely explored, a similar strategy was demonstrated to be viable [47,48], indicating that *Anopheles gambiae* mosquitoes could be transfected orally using RNAi protected in nanoparticles, highlighting the potential of nanocarrier-mediated nucleic acid delivery in insects.

Given that larval molting involves extensive physiological events, doubts arise regarding the persistence of transfected pDNA across successive instars. Thus, it is worth considering whether oral transfection is transitory or, on the contrary, plasmids persist in larvae throughout the successive developmental stages. From a practical point of view, this determines whether continuous administration is required or if a simple, time-limited dose is sufficient for sustained recombinant protein expression. Although the literature on this topic is limited, Turner et al. [41] provided compelling evidence of the ontogenetic persistence of exogenous nucleic acids after insect transfection. As mentioned above, they fed larvae of the lepidopteran *Epiphyas postvittana* with dsRNA and confirmed the persistence of dsRNA throughout ontogeny, which agrees with our observations (Figure 5).

The detection of plasmid-specific sequences in transfected larvae confirms the uptake of plasmid DNA. To the best of our knowledge, no studies have investigated the potential mechanisms underlying pDNA uptake through the lepidopteran gut. The only available evidence for nucleic acid transcytosis is related to dsRNA. A recent study by McGraw et al. [49] identified endocytosis and macropinocytosis in both *Spodoptera frugiperda* Sf9 cells and ex vivo midgut tissue as mechanisms facilitating the transport of dsRNA from the intestinal lumen to the hemolymph.

However, pDNA presence alone does not necessarily imply successful transcriptional activity of the reporter gene, and therefore, the detection and quantification in tissues of specific mRNA is needed to confirm gene transcription, as the results point to (Figure 6A,B). Furthermore, in addition to the quantification of mRNA, the functional expression of the encoded proteins was demonstrated by detecting and quantifying the biological activity of the reporter gene products (the bacterial β-galactosidase enzyme for the pCMVβ plasmid and green fluorescence for the pEGFP-N2 plasmid) (Figure 7A,B). These results demonstrate not only successful oral transfection but also the transcription and translation of the recombinant genes.

When it comes to upscaling the production of any recombinant protein, one of the main challenges is the ability to control the expression level in the chosen biological platform. Interestingly, the dietary inclusion of increasing concentrations of chitosan-nanoparticulated pDNA resulted in the enhanced expression of both reporter genes in *S. exigua* larvae (Figure 6 and Figure 7). These results reinforce the potential of oral transfection as a viable approach, combining ease of administration with a degree of predictability in the expression level of the gene of interest. This aspect is virtually unattainable with most of the current insect transfection methods, which limits their applicability for industrial-scale protein production. According to the results, no saturation effect was observed across the range of pDNA levels tested. Expression levels of both reported genes showed a clear dose-dependent and proportional response, as evidenced by high correlation coefficients (R^2^ values of 0.882 and 0.953 for pCMVβ and pEGFP-N2, respectively) between pDNA concentration in the diet and relative gene expression measured by quantitative PCR. This pattern was mirrored at the protein level, and thus, the biological effects measured for both de novo synthesized recombinant proteins (Figure 7A,B) also exhibited strong dose-dependent relationships (R^2^ values of 0.996 and 0.967 for β-gal activity and green fluorescence, respectively).

These results confirm that oral transfection is feasible in *S. exigua*, provided that pDNA is adequately protected against gut inactivation prior to its incorporation in artificial diets. The nanoparticle formulation carried out in this study not only preserved plasmid integrity and functionality, as evidenced by gene expression and protein activity, but also enabled the modulation of the expression levels by adjusting nanoparticle concentration in the artificial diet. In contrast to viral transfection systems, plasmid-based delivery does not require special biosafety contention measures to prevent uncontrolled infections in large-scale insect rearing facilities. Additionally, plasmid transfection does not induce cell lysis, which is compatible with physiological growing and molting during the life cycle of the insects, at least in *S. exigua*.

It is also worth pointing out that this study did not utilize insect-specific expression vectors. Instead, we employed general eukaryotic plasmids driven by the human cytomegalovirus (CMV) promoter, widely used in mammalian systems. Although the CMV promoter can drive gene expression in insect cells, it is assumed that its activity is generally weaker than that of insect-specific promoters [50]. However, the results evidenced that this choice allowed for consistent measurements of both reporter gene expression and functional protein output, thus serving as a reliable proof of concept, since the level of expression was sufficient for the experimental goals. Nonetheless, it is reasonable to assume that expression yields could be significantly improved by considering insect-specific promoters, such as *polyhedrin* or *p10*, which have been widely used to optimize expression in insect cell systems [2].

Hence, there is no doubt that there exists room for improving the yield of recombinant protein expression in larvae by designing plasmids including insect-specific promoters, which in fact represents an optimization strategy to improve protein expression in insect cell culture.

It is also worth mentioning that this approach may also broaden the range of insect species with potential to be used as expression platforms, provided that they can be reared on artificial diets. In this regard, while Lepidoptera species are frequently used in laboratory-scale transfection studies, they are not yet reared at the industrial scale for the biomass of protein extraction, at least in the European Union. In contrast, mass-reared insect species with significant commercial relevance include members of the orders Coleoptera (e.g., *Tenebrio molitor*), Diptera (e.g., *Hermetia illucens*) and Orthoptera (e.g., *Acheta domesticus*). These species, given their compatibility with artificial diets, represent promising candidates for the further exploration of oral transfection technologies.

## 5. Conclusions

This study provides proof of concept that the oral delivery of plasmid DNA, when protected through nanoencapsulation, enables effective transfection, gene expression, and recombinant protein production in *Spodoptera exigua* larvae. The approach demonstrated dose-dependent transcription and protein activity, showing both the stability of the DNA and predictability of expression levels. Plasmid-based oral delivery offers a simple, non-invasive method that avoids biosafety concerns and is compatible with normal insect growth. While this work relied on general eukaryotic promoters, future optimization using insect-specific regulatory elements could further increase yields. Importantly, the method is not restricted to *S. exigua* and may be extended to other insect species that are already mass reared on artificial diets, including those of high industrial interest. Overall, oral transfection represents a feasible and scalable strategy for exploiting insects as platforms for recombinant protein production.

## Figures and Tables

**Table 1:** Pairs of primers used for the identification of plasmids by conventional PCR.

			Sequence (5′-3′)	
Pair	Plasmid	Product Size (pb)	Forward	Reverse
β-gal1	pCMVβ	682	CTTTCACAGATGTGGATTGG	CCATATGGAAACCGTCGATA
β-gal2	pCMVβ	1361	CAAAGAACTGCTCCTAGTGG	TTCTGCTTCATCATCAGCAGGATA
EGFP1	pEGFP-N2	187	ATCATGGCCGACAAGCAGAA	TCTCGTTGGGGTCTTTGCTC
EGFP2	pEGFP-N2	250	GGACGACGGCAACTACAAGA	TCTCGTTGGGGTCTTTGCTC

**Table 2:** Pairs of primers used in qRT-PCR assays.

Gene	Sequence (5′-3′)	
	Forward	Reverse
*lacZ*	GATGAAGACCAGCCCTTCCC	CCGCCAAGACTGTTACCCAT
*lacZ*	CAGTACATCAATGGGCGTGG	AGTCCCGTTGATTTTGGTGC
*EGFP*	CTGCTGCCCGACAACCAC	TCACGAACTCCAGCAGGAC
*EGFP*	TGCTGCTGCCCGACAACCACTAC	CTTGTACAGCTCGTCCATGCC
*rsp10* ^1^	GGCTACGGTCGACGACTTCCC	GCAGCCTCATGCGGATGTGGAAC
*gadph* ^2^	CTGAGGAGGTCGTGTCATCCG	GATCGATAACGCGGTTGGAGTAGCC
*act* ^3^	GGCTGCCGACATAGACATGCG	GGGTCCTCCACGCGGATCTT

^1^ rsp10: ribosomal protein S10. ^2^ gadph: glyceraldehyde 3-phosphate dehydrogenase. ^3^ act: Spodoptera exigua actin.

## Data Availability

The raw data supporting the conclusions of this article will be made available by the authors on request.
